# Antiosteoporosis effect of tanshinol in osteoporosis animal models: A systematic review and meta-analysis

**DOI:** 10.3389/fphar.2022.937538

**Published:** 2022-08-10

**Authors:** Shen Wang, Yifeng Yuan, Qian Lin, Hang Zhou, Binbin Tang, Yang Liu, Hai Huang, Bocheng Liang, Yingdelong Mao, Kang Liu, Xiaolin Shi

**Affiliations:** ^1^ The Second School of Clinical Medicine, Zhejiang Chinese Medical University, Hangzhou, China; ^2^ Changping District Hospital of Integrated Traditional Chinese and Western Medicine, Beijing, China; ^3^ The Second Affiliated Hospital of Zhejiang Chinese Medical University (Xinhua Hospital of Zhejiang Province), Hangzhou, China

**Keywords:** osteoporosis, tanshinol, systematic review, meta-analysis, animal model

## Abstract

**Background:** Osteoporosis (OP) is an age-related bone disease that has emerged as a worldwide public health concern due to its increasing incidence and high disability rate. Tanshinol [D (+) β-3,4-dihydroxyphenyl lactic acid, TS], a water-soluble component extracted from Salvia miltiorrhiza, has proven to be effective in attenuating OP *in vitro* and *in vivo*. However, there is insufficient evidence to support its clinical application.

**Objective:** This meta-analysis aimed to investigate available OP animal model studies to demonstrate the antiosteoporosis effects of TS in a systematic manner.

**Methods:** Electronic searches of related studies were conducted in the following databases: EMBASE, PubMed, Web of Science, Cochrane Library, Chinese National Knowledge Infrastructure, Chinese VIP Database, Chinese Biomedical Literature Database, and Wanfang. The retrieval date was January 2022, and there were no time or language restrictions. The CAMARADES 10-item quality checklist was utilized to test the risk of potential bias for each study, and modifications were performed accordingly. The primary outcome was bone mineral density (BMD, which included the femur and lumbar spine); and secondary outcomes were parameters for trabecular bone such as bone volume over total volume (BV/TV), trabecular number (Tb.N), trabecular thickness (Tb.Th), trabecular separation (Tb.Sp), conditions of the femur (including bone maximum load and bone elastic load), and markers of bone metabolism (serum osteocalcin, S-OCN).

**Results:** A total of nine studies including 176 rats were chosen for this analysis. Egger’s test revealed the presence of publication bias in various studies regarding the primary outcome. According to this systematic review, TS significantly increased the BMD of the femur (BMD-femur) (*SMD* = 4.40; 95% CI = 1.61 to 7.19; *p* = 0.002, *I* ^*2*^ = 94.6%), BMD of the lumbar spine (BMD-lumbar) (SMD = 6.390; 95% CI = 2.036 to 10.744; *p* = 0.004, *I^2^* = 95.9%), BV/TV (SMD = 0.790; 95% CI = 0.376 to 1.204; *p* = 0.000, *I^2^* = 10.8), Tb.N (SMD = 0.690; 95% CI = 0.309 to 1.071; *p* = 0.000, *I^2^* = 12%), Tb.Th (SMD = 0.772; 95% CI = 0.410 to 1.134; *p* = 0.000, *I^2^* = 32.2%), and S-OCN (SMD = 3.13; 95% CI = 0.617 to 5.65; *p* = 0.015, *I^2^* = 92.3%), while the Tb.Sp level was markedly decreased in OP models in comparison to the controls (SMD = −0.822; 95% CI = −1.207 to −0.437; *p* = 0.000, *I^2^* = 0%). Moreover, TS treatment was associated with a significant improvement of the bone biomechanical indicators, including bone maximum load (SMD = 0.912; 95% CI = 0.370 to 1.455; *p* = 0.001, *I^2^* = 40%) and elasticity load (SM*D* = 0.821; 95% CI = 0.290 to 1.351; *p* = 0.002, *I* ^*2*^ = 0%).

**Conclusion:** Collectively, our findings suggest that TS can improve BMD, bone microarchitecture, bone biomechanics, and S-OCN expression in rats, implying that it could be used clinically in the future.

**Systematic Review Registration:** https://inplasy.com/inplasy-2022-3-0053/, identifier [INPLASY202230053].

## Introduction

Osteoporosis (OP) is a disease characterized by low bone mass and structural degeneration that is becoming more prevalent globally as the elderly population grows. OP can be induced by various factors, including diet, diabetes, and medication-associated side effects. Many complications, such as fractures and chronic pain, have been proven to be associated with osteoporosis and are a considerable burden on the healthcare system. In the United States, it is reported that over 40 million women are diagnosed with low bone density, and 300,000 suffer from hip fractures each year (Black and Rosen, 2016). In addition, the annual osteoporosis-related costs in China have been estimated at $25.43 (95% CI = 23.92–26.95) billion by 2050 (Si et al., 2015).

Several treatments for OP have been shown to be effective, including diet modifications, rehabilitation exercises, and medication. Medication is the most effective treatment option, with others serving as adjuvant therapy. On the other hand, calcium supplements and active vitamin D can only increase bone calcium content while having a limited regulatory effect on bone metabolic balance. Although some medications have been shown to reduce fracture incidence, they are frequently associated with some potential side effects. For instance, bisphosphonates can lead to jaw osteonecrosis (Drake et al., 2008) and severe musculoskeletal pain, while disuzumab use often leads to back and limb pain (Deeks, 2018). In addition, teriparatide use may impair the cardiovascular, central nervous, and endocrine systems and induce other systemic diseases (Lindsay et al., 2016). Therefore, exploring alternative treatment options with a better safety profile is important.

Traditional Chinese medicine (TCM) has been used to treat many diseases in China for a long time. Salvia miltiorrhiza is known to improve blood circulation, remove blood stasis, and relieve pain; as a result, it has been widely used in the treatment of vascular diseases, coronary heart diseases, and musculoskeletal diseases. Tanshinol [D (+)β-3,4-dihydroxyphenyl lactic acid, TS] is a water-soluble component extracted from Salvia miltiorrhiza (Liu, 2019; Wang, 2015). Several *in vivo* and *in vitro* studies indicated that tanshinol has anti-OP effects (Cui et al., 2009; Luo et al., 2016; Yang et al., 2016; Chen et al., 2017; Han and Wang, 2017). However, its clinical application has been limited due to insufficient evidence. Meta-analysis of *in vivo* studies has been found to be useful in determining the efficacy and safety of drugs. To that end, we included animal studies that previously examined the potential effect of TS in OP treatment in our analysis to provide a scientific basis for future clinical trials.

## Materials and methods

### Literature retrieval

Electronic study search was conducted in the following databases: EMBASE, PubMed, Web of Science, Cochrane Library, Chinese National Knowledge Infrastructure, Chinese VIP Database, Chinese Biomedical Literature Database, and Wanfang Database. No time or language restrictions were set, and the retrieval date was January 2022. The search algorithm was adapted according to the different database requirements. For instance, the retrieval strategy for Web of Science was ((((TS=(“Tanshinol”)) OR TS=(Danshensu)) OR TS=(“dan shen su a”)) OR TS=(“Salvianic aid A”)) AND TS=(osteoporosis).

### Inclusion criteria

Animal studies that fulfilled the following conditions were included in our study: 1) experimental groups received tanshinol as monotherapy, while the corresponding control groups were treated with a vehicle or received a placebo such as saline solution; 2) studies with conclusive results; and 3) animals models established using different methods, regardless of species, age, weight, or gender.

### Exclusion criteria

Studies with the following conditions were excluded from the analysis: 1) *in vitro* studies, case reports, clinical trials, reviews, abstracts, comments, and editorials, 2) studies that did not use an acceptable established osteoporosis model, 3) studies with missing data, 4) duplicate publications, and 5) studies in which no outcome indicators were used.

### Outcome measurements

The primary outcome of this study was bone mineral density (BMD, including the femur and lumbar spine), and the secondary outcomes were the static parameters for trabecular bone: bone volume over total volume (BV/TV), trabecular number (Tb.N), trabecular thickness (Tb.Th), trabecular separation (Tb.Sp), biomechanical quality of the femur: bone maximum load, bone elastic load, and serum osteocalcin (S-OCN).

### Data extraction

Data were extracted by two authors independently and reviewed by a third author. The following information was extracted from each study: author name, date of publication, animal species, age, sex, body weight, sample size, OP modeling methods, anesthetics method used, the treatment protocol for control and experimental groups, and primary and secondary outcomes. We extracted the mean and standard deviation (SD) for continuous variables. If different doses of tanshinol were used, only data with the highest dose were collected. Authors of these publications were contacted to obtain relevant data where necessary.

### Data analysis

Data analyses were performed using the Stata software (Stata SE, version 16). When significant heterogeneity (*I* ^*2*^ ≥ 50%) was detected, sensitivity analysis was performed to identify the possible cause. A fixed-effects model was used when heterogeneity was not detected (*I* ^*2*^ < 50%) or when the effects of significant clinical heterogeneity were excluded. Moreover, Egger’s test was conducted to investigate the effect of publication bias. We calculated the pooled estimate as a standard mean difference (*SMD*) with a 95% confidence interval (*CI*) for continuous outcomes.

## Results

### Literature selection

**FIGURE 1:**
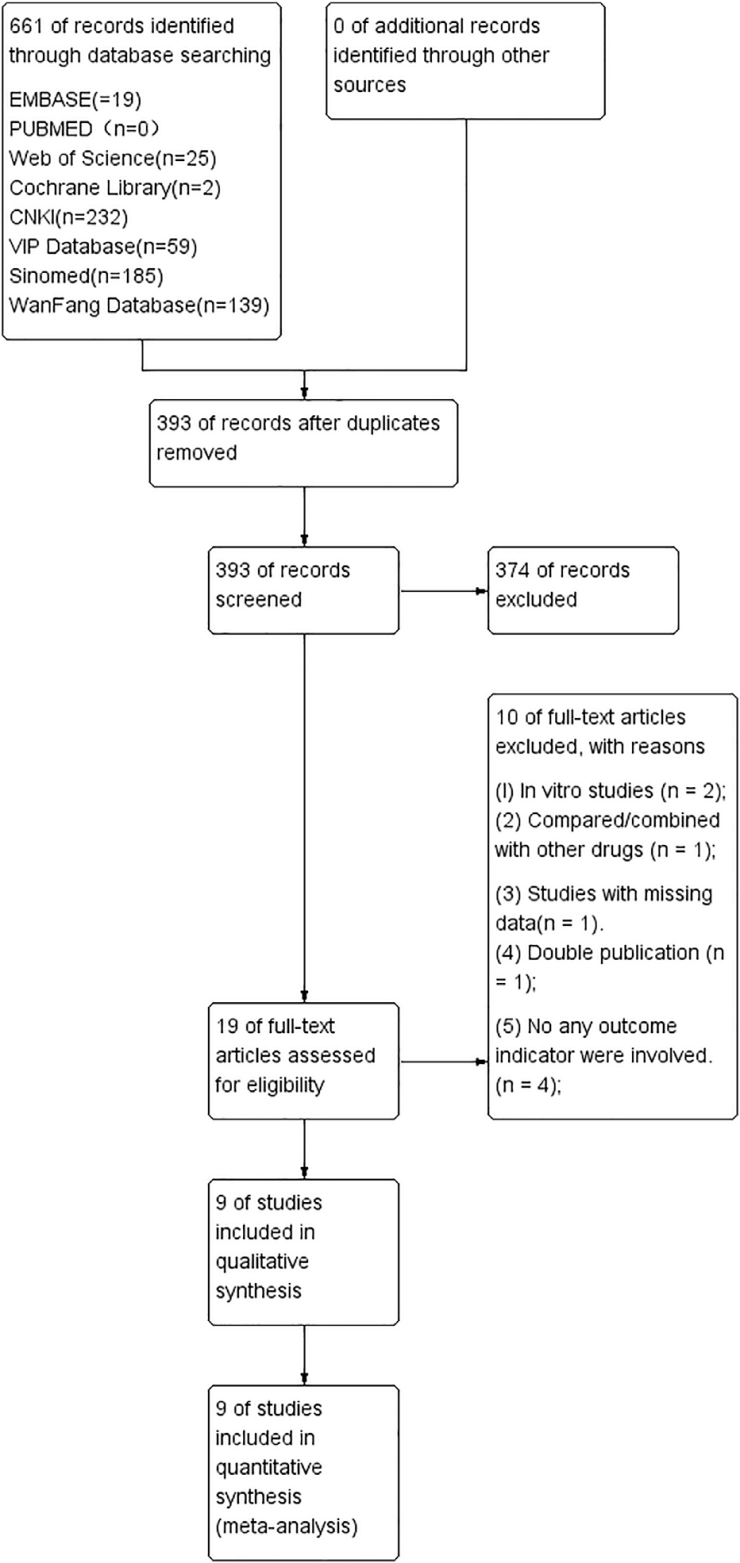
The study’s screening workflow.

A total of 661 articles were identified after searching eight databases, and 268 were excluded due to duplication. After reviewing the abstract, another 374 studies were eliminated. The remaining 19 studies were read in full, and ten reports were excluded because of the following: TS was compared/combined with other drugs; there was no control group; and/or there was data duplication. Eventually, nine studies were selected for this meta-analysis, including two published in English (Yang et al., 2016; Lai et al., 2021) and seven published in Chinese (Zhang et al., 2011; Chen J, 2015; Qu et al., 2016; SY, 2016; Wang, 2017; Du, 2018; Sang, 2019) (Figure 1).

### Basic information of the included studies

Table 1 shows the specifics of the nine studies that were selected. All studies were published between 2011 and 2021, and a total of 176 subjects were enrolled, with 88 in the experimental group and 88 in the control group. Sample sizes of enrolled studies ranged from 16 to 24 (median = 20). Sprague–Dawley (SD) rats were used for all studies, and seven studies used female (75%) murine models (Zhang et al., 2011; Chen J, 2015; Qu et al., 2016; SY, 2016; Yang et al., 2016; Wang, 2017; Sang, 2019), while two studies used male (25%) murine models (Du, 2018; Lai et al., 2021). Rats in five studies were treated with oral prednisone acetate (PA, 5 mg–6 mg/kg.d, 14–16 weeks) (Chen J, 2015; SY, 2016; Yang et al., 2016; Wang B, 2017; Lai et al., 2021), two with bilateral oophorectomy (Zhang et al., 2011; Qu T, 2016), one with oral levothyroxine (0.25 mg/kg.d, 12 weeks) (Du, 2018), and one with oral retinoic acid (70 mg/kg/d, 2 weeks) (Sang, 2019). The rats in all of the enrolled studies received intragastrical tanshinol at doses ranging from 12.5 mg/kg.d to 50 mg/kg.d (Zhang et al., 2011; Chen J, 2015; Qu T, 2016; SY, 2016; Yang et al., 2016; Wang B, 2017; Du, 2018; Sang, 2019; Lai et al., 2021). In terms of the primary outcome, BMD-femur was measured in five studies (Qu T, 2016; SY, 2016; Wang B, 2017; Du, 2018; Sang L, 2019), while four studies quantified BMD-lumbar (SY, 2016; Sang L, 2019; Wang B, 2017; Qu T, 2016). In addition, the BV/TV value was measured in five studies (Chen J, 2015; Yang et al., 2016; SY, 2016; Du, 2018; Lai et al., 2021), Tb.N in six studies (Zhang et al., 2011; Chen J, 2015; SY, 2016; Yang et al., 2016; Du, 2018; Lai et al., 2021), Tb.Th in six studies (Zhang et al., 2011; Chen J, 2015; SY, 2016; Yang et al., 2016; Du, 2018; Lai et al., 2021), Tb.Sp in six studies (Zhang et al., 2011; Chen J, 2015; SY, 2016; Yang et al., 2016; Du, 2018; Lai et al., 2021), bone maximum load in four studies (Qu et al., 2016; SY, 2016; Du, 2018; Lai et al., 2021), bone elastic load in four studies (SY, 2016; Yang et al., 2016; Du, 2018; Lai et al., 2021), and serum osteocalcin (S-OCN) in four studies (Qu et al., 2016; SY, 2016; Yang et al., 2016; Du, 2018).

**TABLE 1:** The details of included studies.

Study (year)	Animals	Weight	Age	Anesthetic	Sample size (n = experimental/control group)	Experimental group	Control group	Outcome indicators	Duration
Zhang et al. (2011)	Female SD rats	299 ± 20 g	6 months	NG	8/8	TS (12.5 mg/kg.d P.O)+OVX	Distilled water + OVX	(4) (5) (6)	90 days
Chen (2015)	Female SD rats	300 ± 20 g	7 months	NG	10/10	TS (50 mg/kg.d,P.O)+PA (6 mg/kg.d,qd,P.O)	PA (6 mg/kg.d,qd,P.O)<	(3) (4) (5) (6)	14 weeks
Luo SY (2016)	Female SD rats	300 ± 10 g	7 months	Peritoneal injection of sodium pentobarbital	10/10	TS (50 mg/kg.d,P.O)+PA (6 mg/kg.d,qd,P.O)	Distilled water + PA (6 mg/kg.d,qd,P.O)	(1) (2) (3) (4) (5) (6) (7) (8) (9)	14 weeks
Qu et al. (2016)	Female SD rats	300 ± 20 g	5 months	Peritoneal injection of sodium pentobarbital	10/10	TS 12.5 mg/kg.d P.O + OVX (2 weeks after modeling)	Distilled water + OVX (2 weeks after modeling)	(1) (2) (7) (9)	90 days
Yang YJ et al. (2016)	Female SD rats	200–250 g	4 months	Peritoneal injection of sodium pentobarbital	8/8	TS (16 mg/kg.d,P.O)+PA (5 mg/kg.d,qd,P.O)	PA (5 mg/kg.d,qd,P.O)	(3) (4) (5) (6) (8) (9)	14 weeks
Wang (2017)	Female SD rats	240 ± 10 g	4 months	NG	10/10	TS (30 mg/kg.d,P.O)+PA (5 mg/kg.d,qd,P.O)	PA (5 mg/kg.d,qd,P.O)	(1) (2)	14 weeks
Du (2018)	Male SD rats	325 ± 25 g	3 months	Peritoneal injection of sodium pentobarbital	8/8	TS (25 mg/kg.d,P.O)+levothyroxine (0.25mg/k g.d,qd,P.O)	Levothyroxine (0.25mg/k g.d,qd,P.O) +NS(5 ml/kg.d,qd,P.O)for 12 weeks	(1) (3) (4) (5) (6) (7) (8) (9)	3 months
Sang (2019)	Female SD rats	220–260 g	16 weeks	Peritoneal injection of chloral hydrate	12/12	TS (30 mg/kg.d,P.O) after Modeling (tretinoin 70 mg/kg/d,qd,P.O) for 2 weeks)	5%CMC-Na (5 ml/kg.d,qd,P.O) after modeling (tretinoin 70 mg/kg/d,qd,P.O) for 2 weeks)	(1) (2)	14 weeks
Lai WX et al. (2021)	Male SD rats	396.4 ± 28.55 g	4 months	Peritoneal injection of chloral hydrate	12/12	TS (25 mg/kg.d,qd,P.O)+PA (6 mg/kg.d,qd,P.O)	PA (6 mg/kg.d,qd,P.O)	(3) (4) (5) (6) (7) (8) (9)	16 weeks

(1) BMD-femur, (2) BMD-lumbar, (3) BV/TV, (4) Tb.N, (5) Tb.Th, (6) Tb.Sp, (7) bone maximum load, (8) bone elastic load, and (9) S-OCN.

NG, not given the specific name of anesthetics; OVX, ovariectomized; SD, Sprague–Dawley; PA, prednisone acetate.

### Risk of bias

The risk of bias for each study was tested using the CAMARADES 10-item quality checklist (Macleod et al., 2004) (Table 2). Modifications were implemented where needed (Bao et al., 2018):1) blinded induction of model, 2) use of anesthetic without significant protective and toxic effects on bones. The quality score of studies ranged from 3 to 6 (mean ± SD: 4.5 ± 0.83).

**TABLE 2:** Risk of bias of the included studies.

Study (year)	(1)	(2)	(3)	(4)	(5)	(6)	(7)	(8)	(9)	(10)	Total
Zhang et al. (2011)	√	√	√						√		4
Chen (2015)	√	√	√						√		4
Luo SY (2016)		√	√			√			√		4
Qu et al. (2016)	√	√	√			√			√		5
Yang YJ et al. (2016)	√	√	√			√			√	√	6
Wang (2017)	√	√	√						√		4
Du (2018)		√	√			√			√		4
Sang (2019)	√		√			√					3
Lai WX et al. (2021)	√	√	√			√			√		5

(1) publication in a peer-reviewed journal; (2) statement of control of temperature; (3) randomization of treatment or control; (4) blinded induction of model; (5) blinding of outcome assessment; (6) use of anesthetic without proven protective measures that may have toxic effects on bones; (7) appropriate animal model (aged, diabetic, or hypertensive); (8) sample size estimation; (9) compliance with animal welfare regulations; (10) declaration of any potential conflict of interest.

### BMD-femur

**FIGURE 2:**
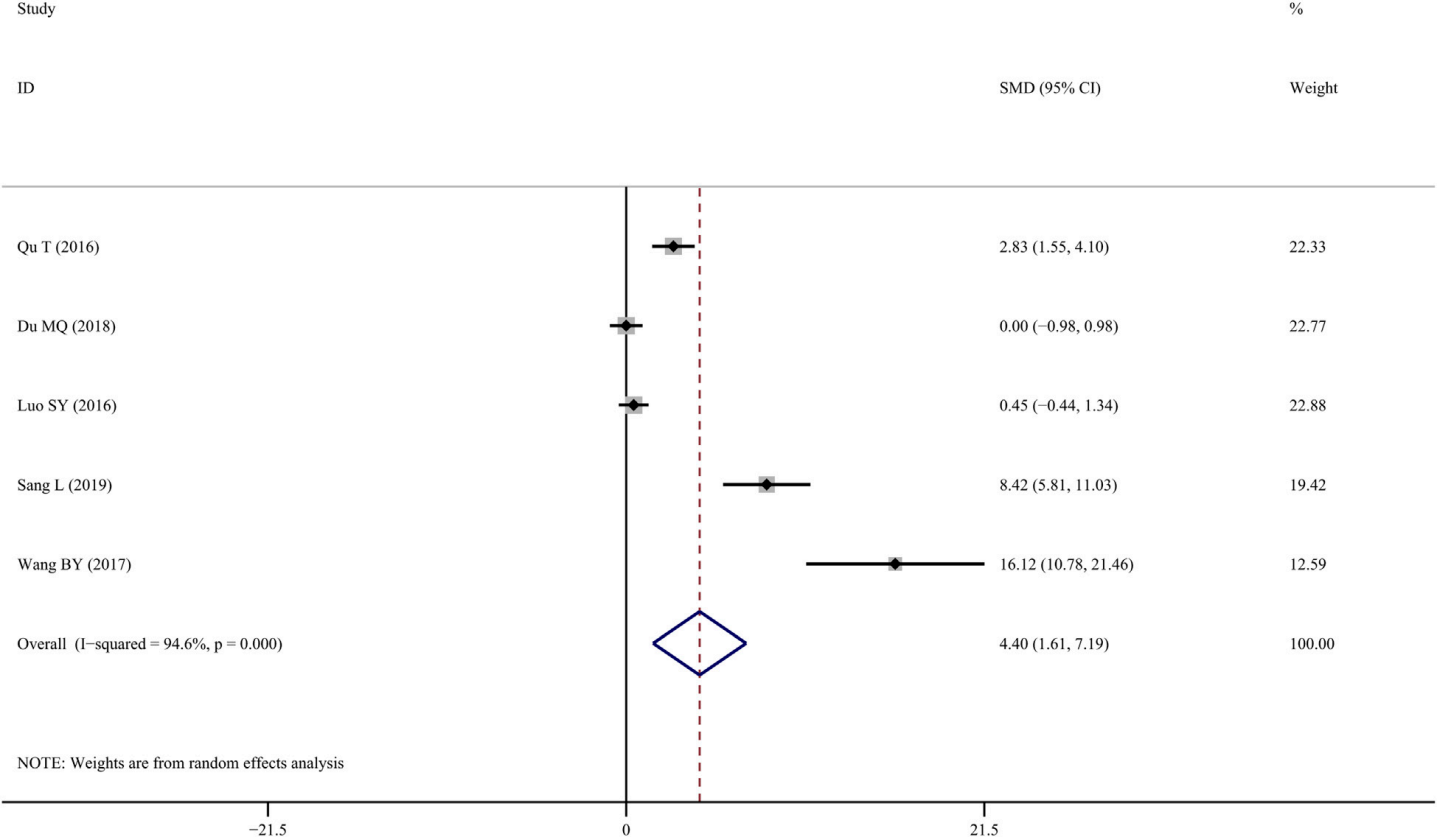
Effect of tanshinol on BMD-femur change in osteoporosis rats.

Five studies (Qu et al., 2016; SY, 2016; Wang, 2017; Du, 2018; Sang, 2019) reported on BMD-femur and revealed that TS treatment was significantly associated with improved BMD values when compared to controls (*SMD* = 4.40; 95% CI = 1.61 to 7.19; *p* = 0.002, heterogeneity *χ* ^*2*^ = 73.92, *df* = 4, *p = 0.000*, *I* ^*2*^ = 94.6%, Figure 2). The random-effect model was chosen given the significant heterogeneity among the included studies. Metaregression was not performed because only a small number of studies were included.

### 3.5 BMD-lumbar

**FIGURE 3:**
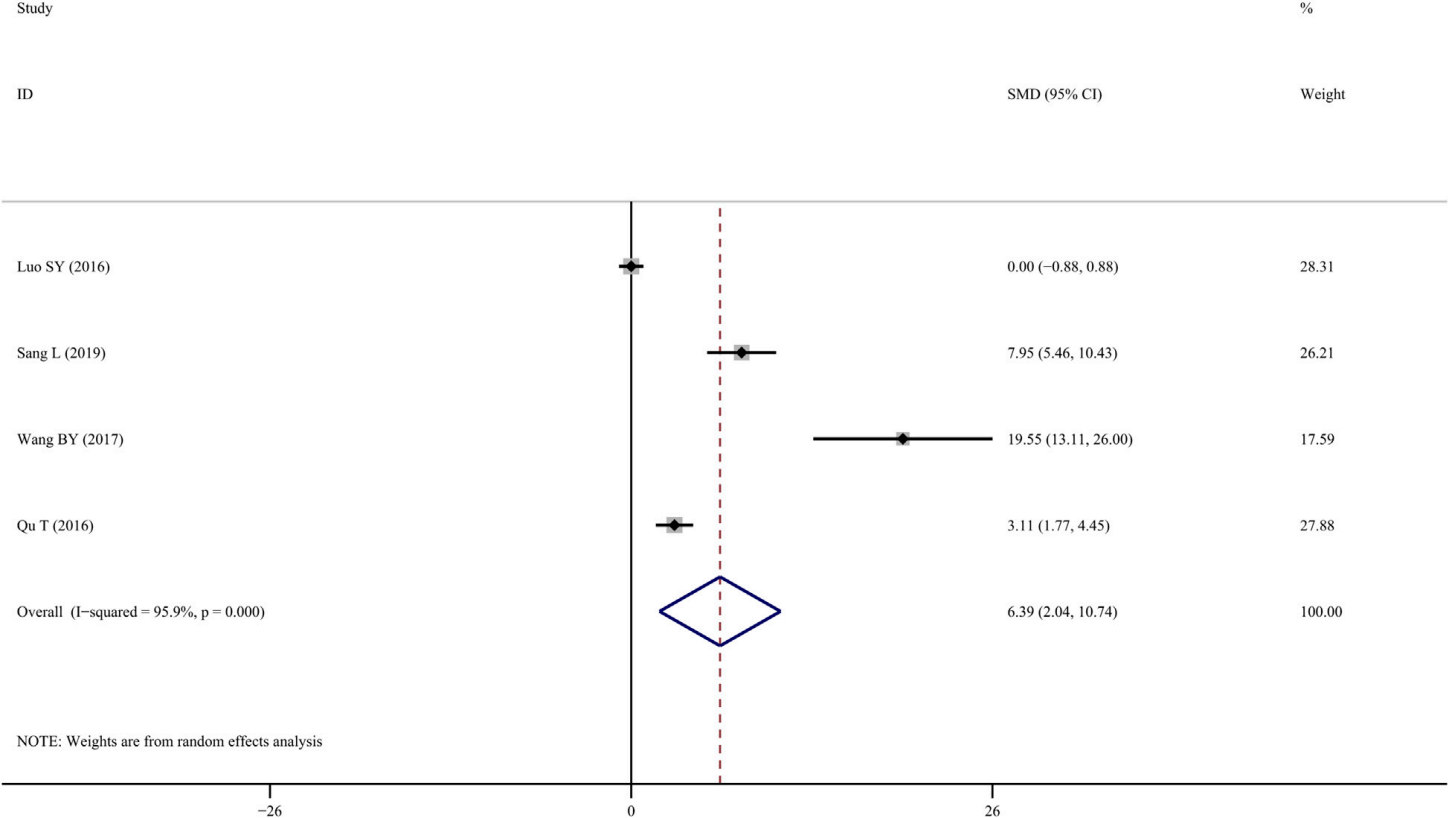
Effect of tanshinol on BMD-lumbar change in OP rats.

Four studies (SY, 2016; Du, 2018; Lai et al., 2021) investigated the efficacy of TS treatment on BMD-lumbar and discovered that TS could significantly enhance BMD values when compared to the control group (*SMD* = 6.390; 95% CI = 2.036 to 10.744; *p* = 0.004, heterogeneity *χ* ^*2*^ = 72.51, *df* = 3, *p* = 0.0001, *I* ^*2*^ = 95.9%, Figure 3). Similarly, the random-effect model was applied due to notable heterogeneity, while metaregression was not performed due to the small sample size.

## The BMD-Related index of femur and tibia under Micro-CT

### BV/TV

**FIGURE 4:**
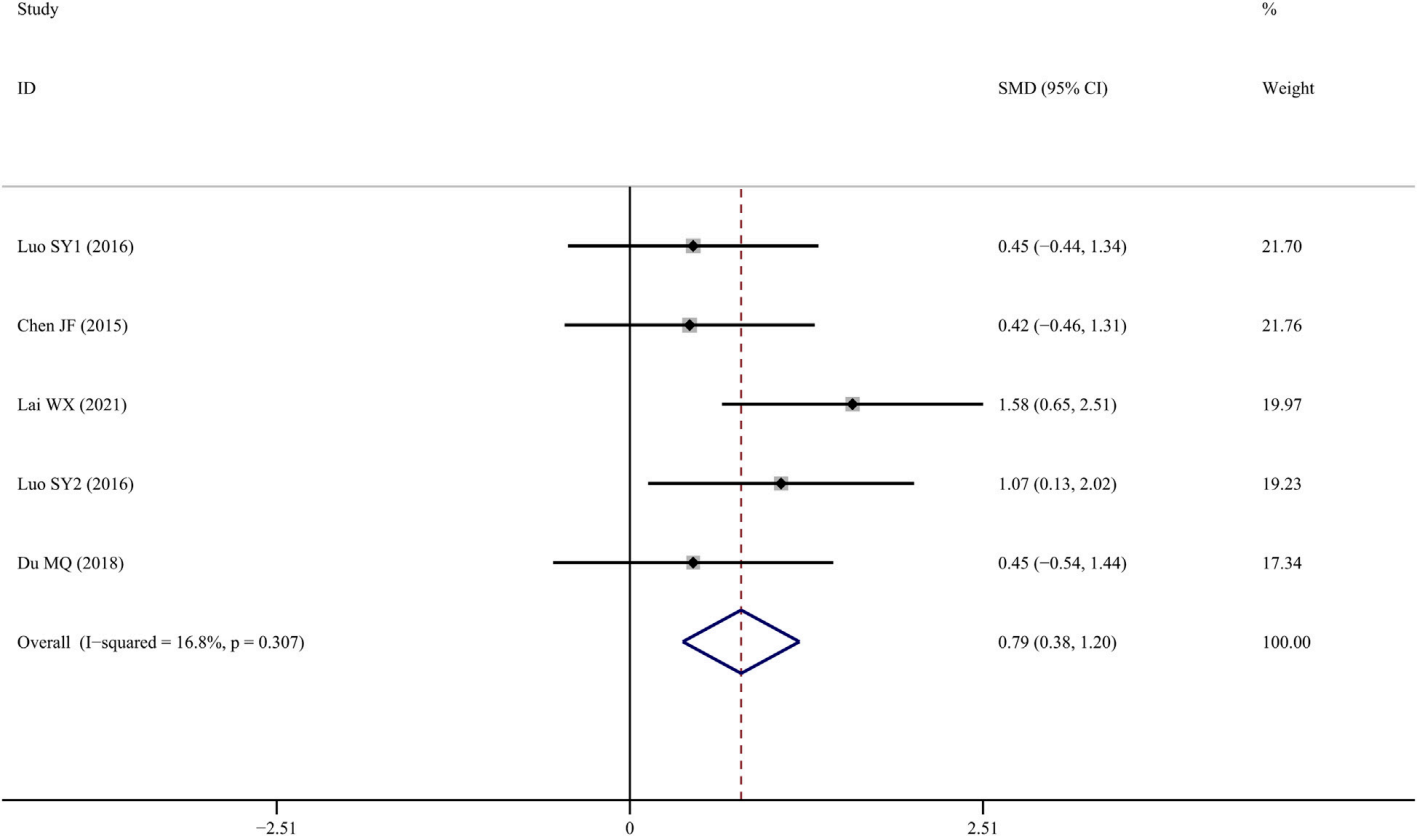
Effect of tanshinol on BV/TV change in OP rats.

Five studies (Chen J, 2015; SY, 2016; Yang et al., 2016; Du, 2018; Lai et al., 2021) measured BV/TV following TS administration, and the results indicated that the TS treatment group showed a significant improvement compared to the control group (*SMD* = 1.192; 95%*CI* = 0.357 to 2.027; *p* = 0.005, heterogeneity *χ* ^*2*^ = 19.71, *df* = 5, *p* = 0.001, *I* ^*2*^ = 74.6%). Sensitivity analyses identified that the source of the heterogeneity was mainly from one study (Yang et al., 2016), and the *I^2^* value was reduced to 10.8% when this study was eliminated. The fixed-effects model revealed that TS treatment increased BV/TV significantly more than control intervention (*SMD* = 0.790; 95% CI = 0.376 to 1.204; *p* = 0.000, heterogeneity *χ* ^*2*^ = 4.81, *df* = 4, *p* = 0.307, Figure 4).

### Tb.N

**FIGURE 5:**
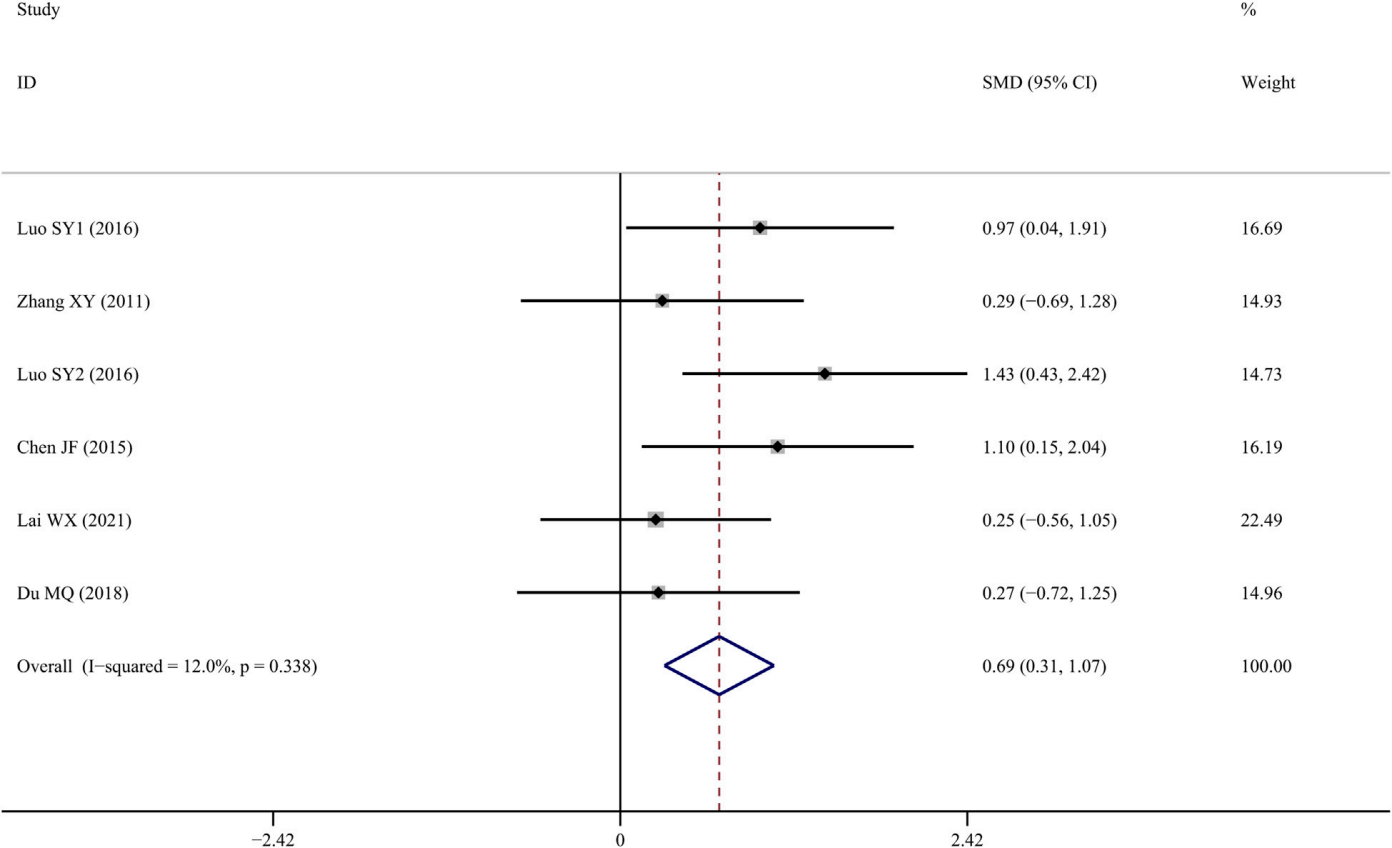
Effect of tanshinol on Tb.N change in OP rats.

Tb.N was reported in six studies (Zhang et al., 2011; Chen J, 2015; SY, 2016; Yang et al., 2016; Du, 2018; Lai et al., 2021), and the results indicated that TS treatment could significantly improve Tb.N values when compared to the control group (*SMD* = 1.096; 95% CI = 0.253 to 1.938; *p* = 0.011, heterogeneity χ^2^ = 26.90, df = 6, *p* = 0.000, *I* ^*2*^ = 77.7%). Similarly, the source of the heterogeneity originated from one study (Yang et al., 2016), and the *I^2^* value was reduced to 12% after removing that article. According to the fixed-effects model, TS significantly increased Tb.N values when compared to controls (*SMD* = 0.690; 95% CI = 0.309 to 1.071; *p* = 0.000, heterogeneity *χ* ^*2*^ = 5.68, *df* = 5, *p* = 0.338, Figure 5).

### Tb.Th

**FIGURE 6:**
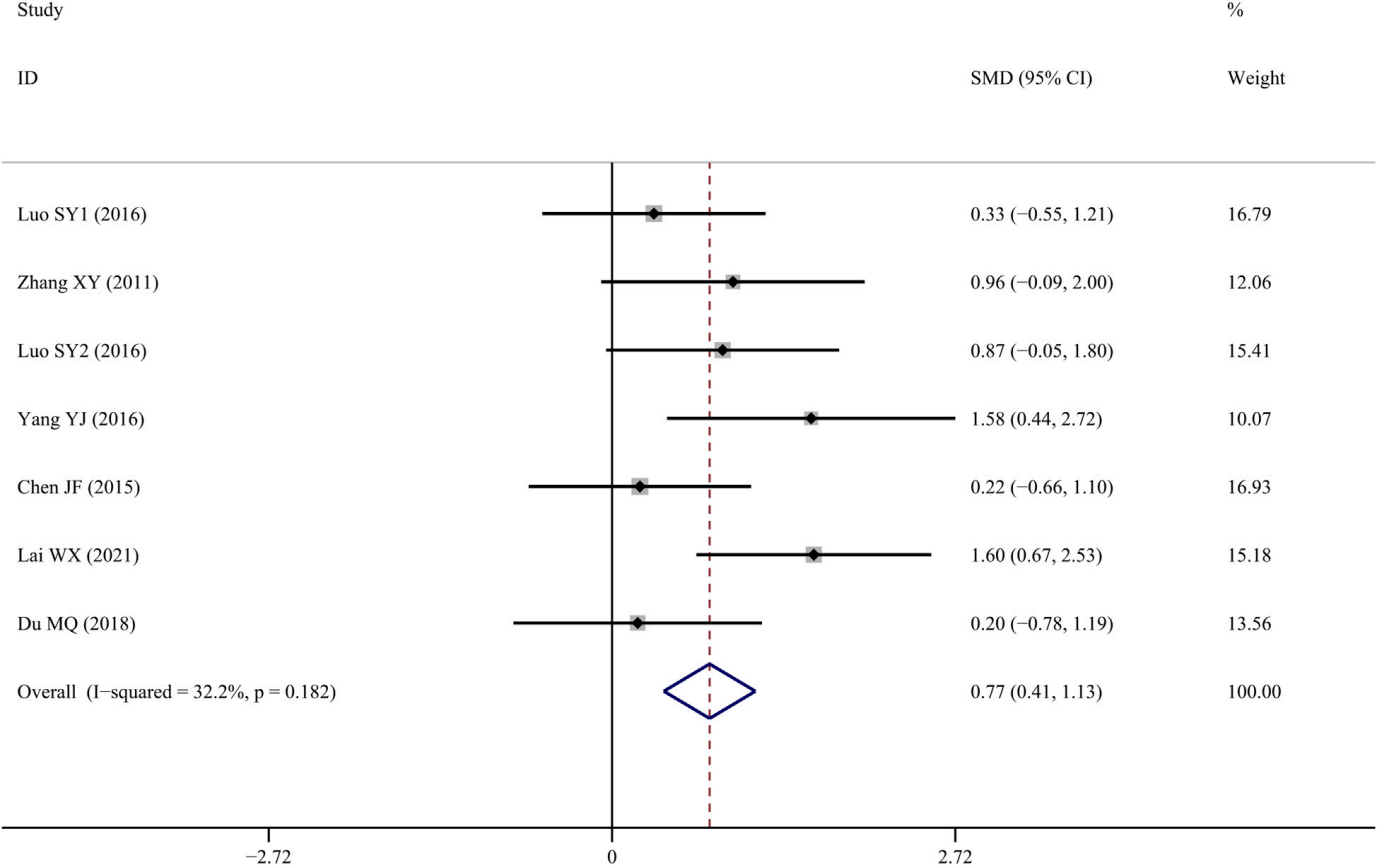
Effect of tanshinol on Tb.Th change in OP rats.

Tb.Th data reported form six studies (Zhang et al., 2011; Chen J, 2015; SY, 2016; Yang et al., 2016; Du, 2018; Lai et al., 2021) indicated that TS treatment was associated with a significant improvement when compared to controls (*SMD* = 0.772; 95% CI = 0.410 to 1.134; *p = 0.000*, heterogeneity *χ* ^*2*^ = 8.85, *df* = 6, *p* = 0.182, *I* ^*2*^ = 32.2%, Figure 6).

### Tb.Sp

**FIGURE 7:**
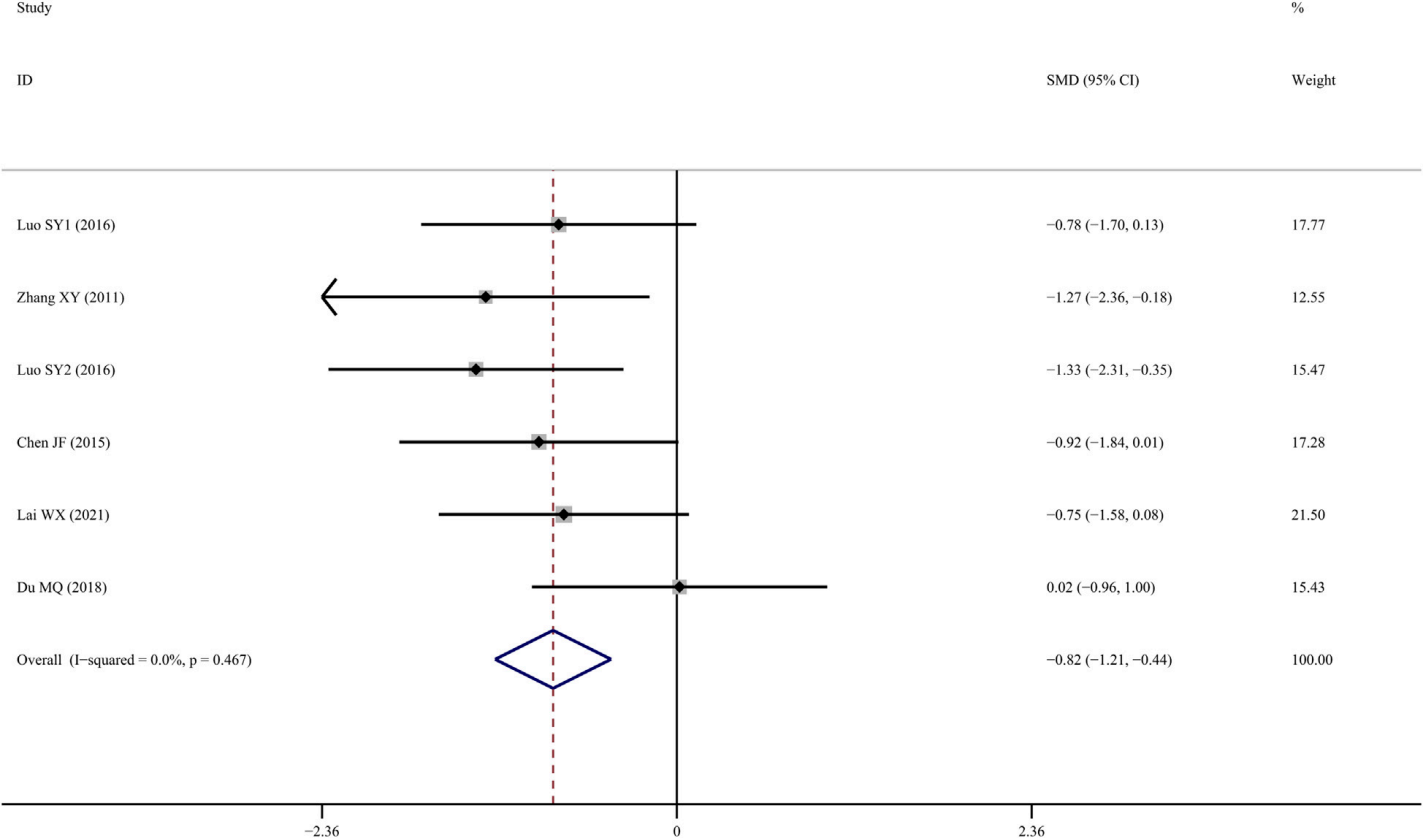
Effect of tanshinol on Tb.Sp change in OP rats.

Tb.Sp results from six studies (Zhang et al., 2011; Chen J, 2015; SY, 2016; Yang et al., 2016; Du, 2018; Lai et al., 2021) suggested that TS treatment was associated with a significant decline when compared to the control group (*SMD* = −1.137; 95% CI = −1.834 to −0.441; *p* = 0.001, heterogeneity *χ* ^*2*^ = 19.11, *df* = 6, *p* = 0.04, *I* ^*2*^ = 68.6%). Sensitivity analyses revealed that the source of the heterogeneity was primarily from one study (Yang et al., 2016), with the *I^2^* value dropping to 0% when that study was removed. According to the fixed-effect model, TS administration significantly reduced Tb.Sp when compared to the control group (*SMD* = −0.822; 95% CI = −1.207 to −0.437; *p = 0.000*, heterogeneity *χ* ^*2*^ = 4.59, *df* = 5, *p* = 0.467, Figure 7).

## Bone biomechanical indicator

### Bone maximum load

**FIGURE 8:**
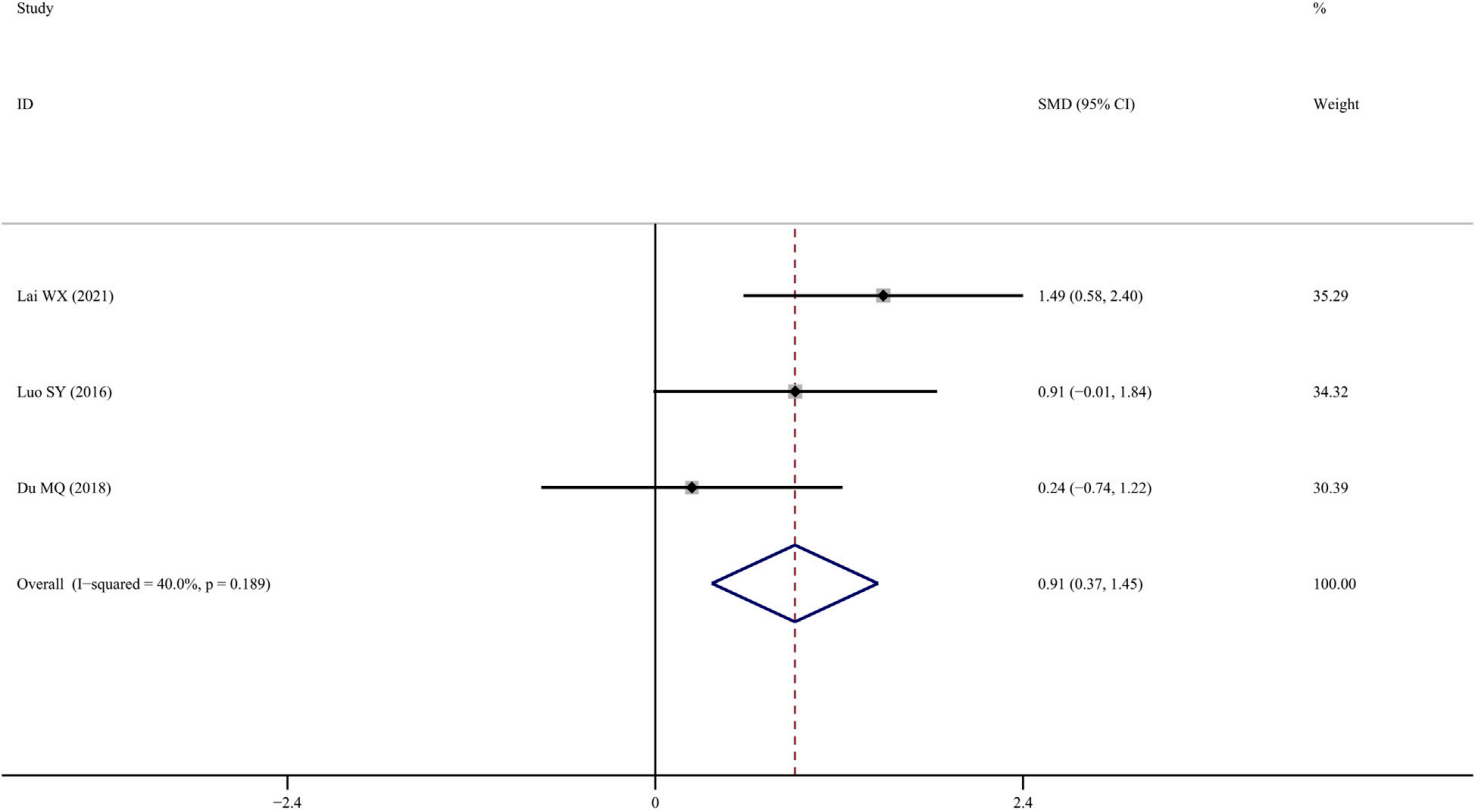
Effect of tanshinol on femur bone maximum load change in OP rats.

Four studies (Chen J, 2015; Qu et al., 2016; Yang et al., 2016; Du, 2018) examined femur bone maximum load, and the results indicated that TS treatment could significantly improve it in comparison to the control group (*SMD* = 1.292; 95% CI = 0.360 to 2.224; *p* = 0.007, heterogeneity *χ* ^*2*^ = 10.27, *df* = 3, *p* = 0.016, *I* ^*2*^ = 70.8%). The source of the heterogeneity was mainly from one study (Qu et al., 2016), and the *I* ^*2*^ value was reduced to 40% when this study was removed. The fixed-effects model indicated that TS treatment could significantly increase femur bone maximum load compared to the control group (*SMD* = 0.912; 95% CI = 0.370 to 1.455; *p* = 0.001, heterogeneity *χ* ^*2*^ = 3.33, *df* = 2, *p* = 0.189, Figure 8).

### Bone elastic load

**FIGURE 9:**
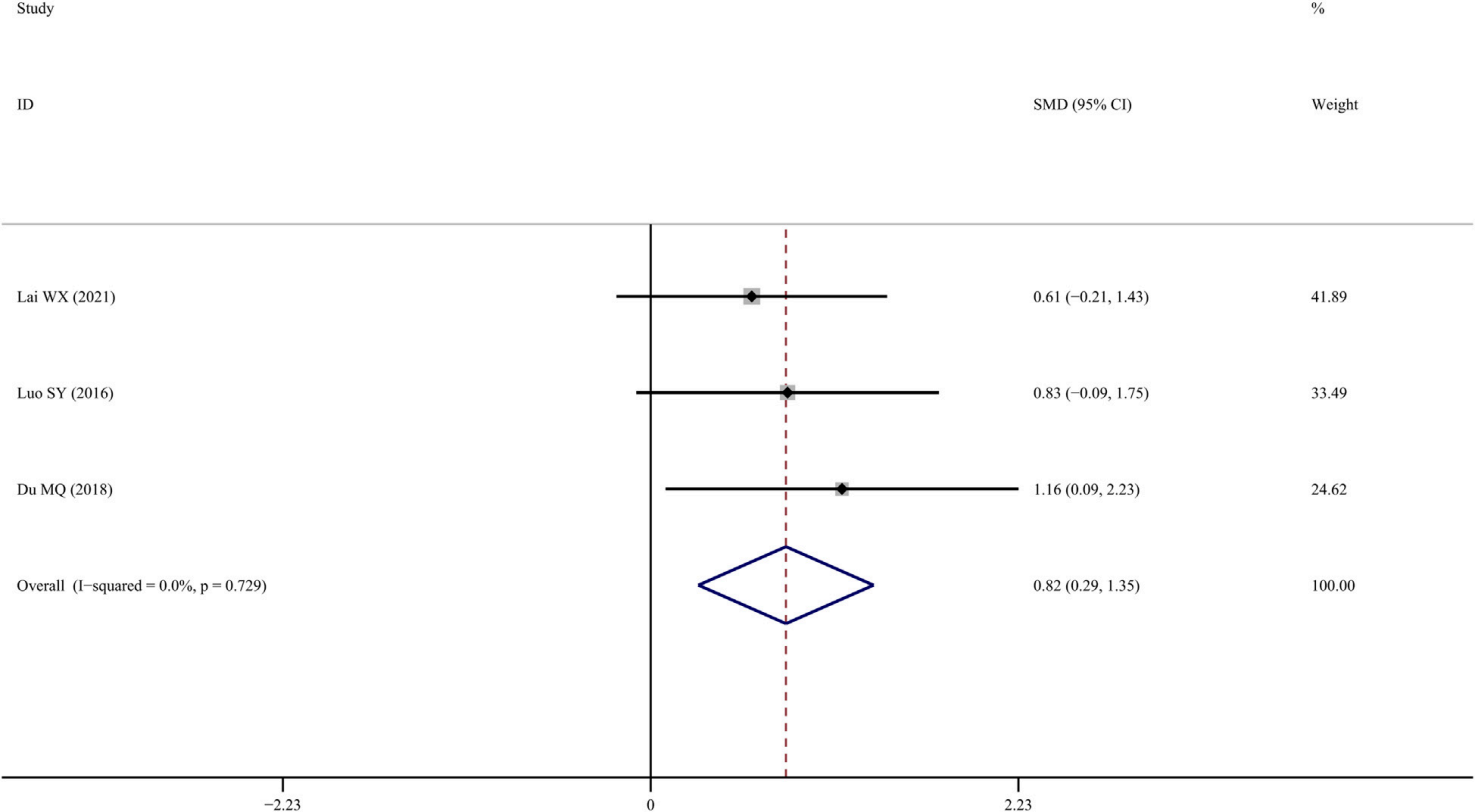
Effect of tanshinol on femur bone elastic load change in OP rats.

Four studies (SY, 2016; Du, 2018; Lai et al., 2021) reported findings on femur bone elastic load, indicating that TS treatment was associated with a significant improvement compared to the control group (SMD = 1.496; 95% CI = 0.332 to 2.660; *p* = 0.01, heterogeneity χ^*2*^ = 13.68, *df* = 3, *p* = 0.003, *I* ^*2*^ = 78.1%). The source of the heterogeneity was mainly generated by one study (Yang et al., 2016), and the *I* ^*2*^ value was reduced to 0% when this article was removed. The fixed-effects model indicated that TS significantly increased femur bone elastic load compared to the controls (*SMD* = 0.821; 95% CI = 0.290 to 1.351; *p* = 0.002, heterogeneity *χ* ^*2*^ = 0.63, *df* = 2, *p* = 0.729, Figure 9).

### S-OCN

**FIGURE 10:**
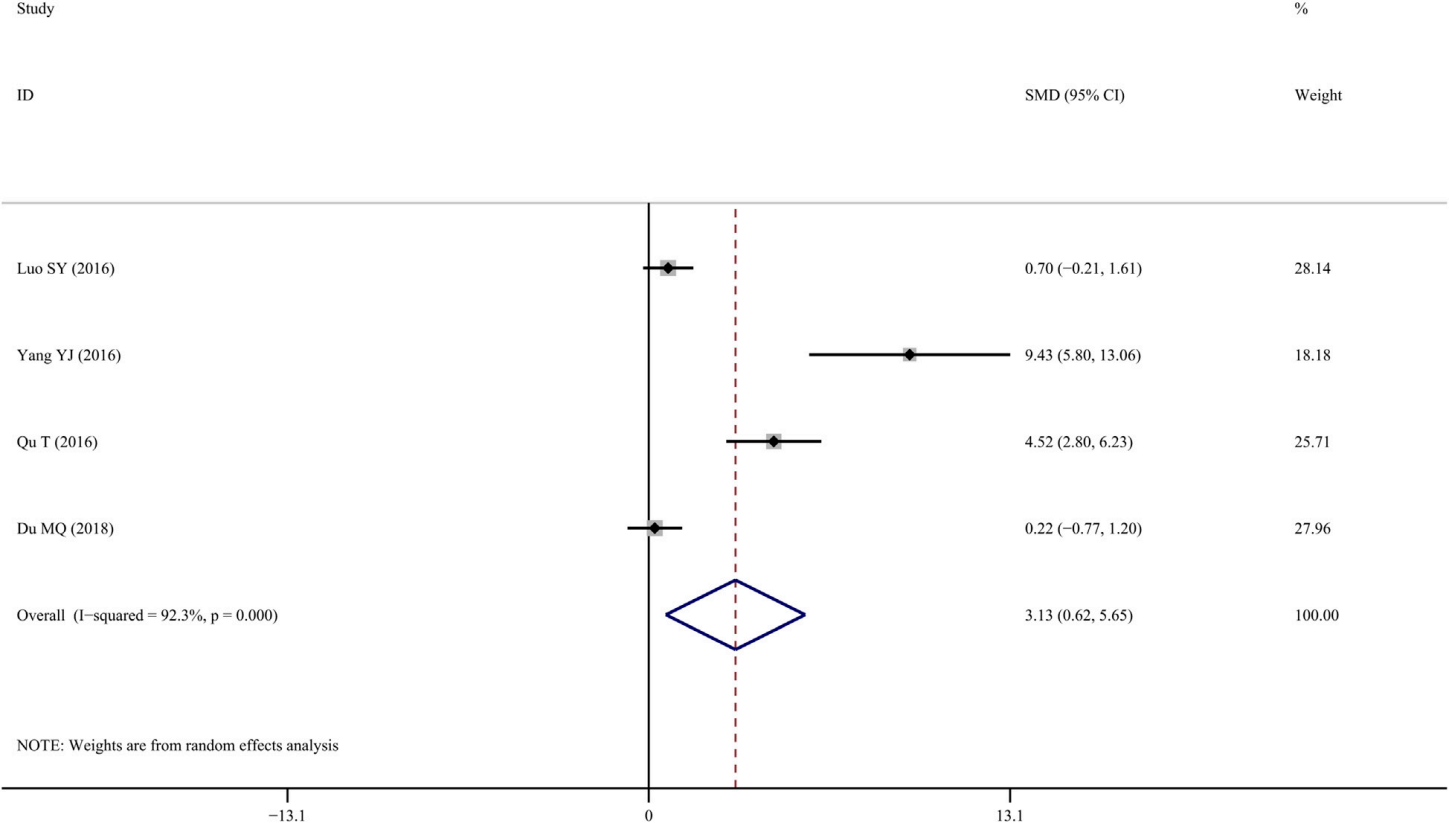
Effect of tanshinol on S-OCN change in OP rats.

Four studies (Qu et al., 2016; SY, 2016; Yang et al., 2016; Du, 2018) reported S-OCN results in the femur, and results indicated that TS treatment was associated with a significant improvement compared with the control group (*SMD* = 3.13; 95% CI = 0.617 to 5.65; *p* = 0.015, heterogeneity *χ* ^*2*^ = 39.1, *df* = 3, *p* = 0.000, *I* ^*2*^ = 92.3%, Figure 10).

### Publication bias and sensitivity analysis

**FIGURE 11:**
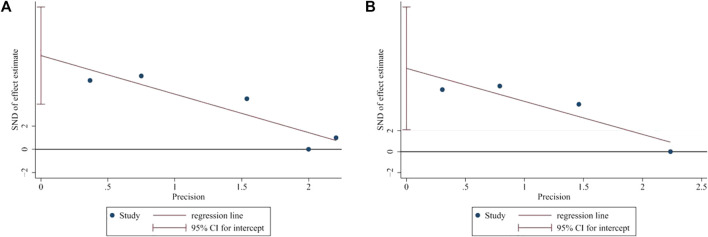
Publication bias. **(A)** Egger’s test of BMD-femur. *p* = 0.009, t = 6.14. **(B)** Egger’s test of BMD-lumbar. *p* = 0.028, t = 5.84. Both **(A)** and **(B)** indicated that there was publication bias.

Egger’s test was applied to assess the potential publication bias in this meta-analysis and identified several publication biases (BMD-femur, *p* = 0.009; BMD-lumbar, *p* = 0.028) (Figure 11). Sensitivity analyses were also conducted by omitting each study, and no obvious effect was found (supplemental material, Figure 12).

**FIGURE 12:**
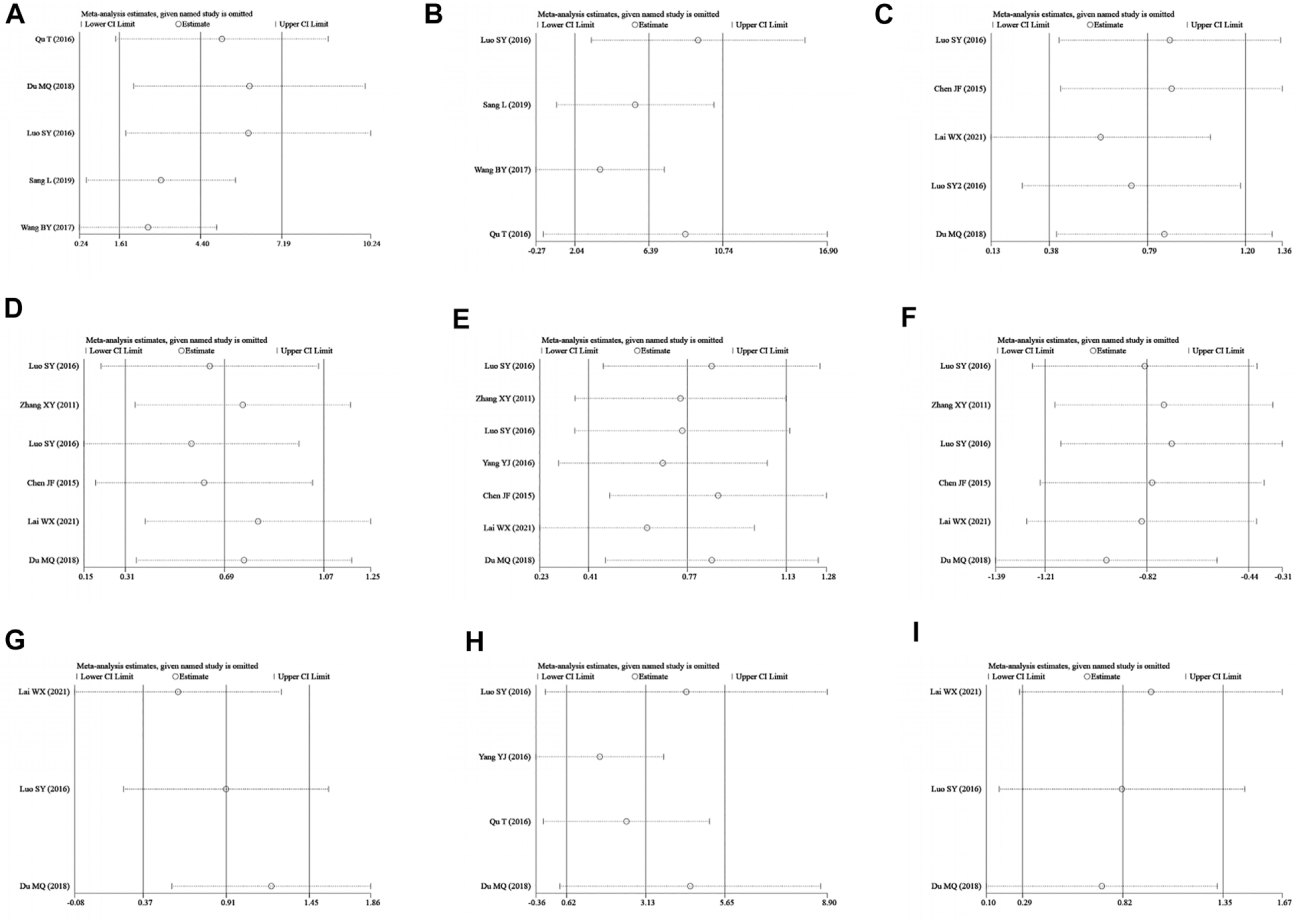
Sensitivity analysis. **(A–I)** represent the sensitivity of BMD-femur, BMD-lumbar, BV/TV, Tb.N, Tb.Th, Tb.Sp, bone max load, S-OCN, and bone elastic, respectively. The sensitivity analysis was conducted by omitting single studies one by one, and no study with critical influence was found.

## Discussion

To the best of our knowledge, this is the first preclinical systematic review and meta-analysis examining TS’s efficacy on osteoporosis. Nine studies with 168 rats were included in this analysis and indicated that the TS treatment significantly increased BMD, BV/TV, Tb.N, Tb.Th, and S-OCN, while reducing Tb.Sp in OP animal models. Moreover, TS treatment was associated with significantly improved bone biomechanical indicators, including bone maximum load and elasticity load.

Notably, the majority of previous meta-analyses examining the effect of TCM herbal components focused on serological indicators and BMD measurements using dual-energy X-ray absorptiometry (DXA) (Fu et al., 2014; Xu et al., 2016; Wu et al., 2020; Yang et al., 2020), while relatively few studies examined the bone structure under micro-CT (Lin et al., 2021; Zhu et al., 2021). Increasing evidence suggests that DXA has some limitations (Hahn et al., 1992). For instance, when measuring low-density bone, such as osteoporotic bone or osteophyte tissue, the DXA approach is associated with errors as high as 30% (Bolotin and Sievänen, 2001, 2001; Warden et al., 2005). However, such shortcomings can be avoided using micro-CT (Laib et al., 2000; Mittra et al., 2005). Furthermore, this technique enables quantitative analysis, which allows for multifaceted comparisons, particularly in small animals (Du et al., 2007; Brouwers et al., 2008). Taken together, using a micro-CT–based technique in BMD measurements can help reveal the geometric features of the trabecular bone and allow for more accurate data analysis.

S-OCN plays an important role in regulating calcium homeostasis and bone metabolism (Seibel, 2000). The level of S-OCN was measured in most animal studies. Several studies have revealed that there are factors that can influence OC values in blood serum, particularly oxalate and fluoride, which are present in anticoagulants (Power and Fottrell, 1991). OCN is highly unstable *in vitro* and should be separated immediately after collection and stored at −70 °C without repeated freezing and thawing since OCN immunoreactivity can be reduced by 40% after repeated freeze–thaw cycles (Power and Fottrell, 1991; Woitge and Seibel, 1999). Moreover, some studies have shown that serum OCN levels are susceptible to many factors, such as circadian rhythm, menstrual cycle, age, ethanol intake, season, and hormone replacement therapy (Gundberg et al., 1985; Thomsen et al., 1989; Nielsen et al., 1990). The large difference in S-OCN levels among enrolled studies may be attributed to the aforementioned factors, which suggest the need to strengthen the training of experimental operators.

As we all know, OP has become a major public health concern, particularly in recent years, due to its rising incidence and disability rates. Bone homeostasis is delicately maintained by a dynamic balance of osteoblasts and osteoclasts. The pathogenic factors of OP are frequently caused by excessive osteoclast activity, which destroys the structure of the trabecular bone and reduces the biomechanical properties of bone tissues. Mounting evidence demonstrates that active ingredients extracted from natural plants could influence these processes and exert a protective role. Existing studies have proven that TS can reduce the loss of bone components and stimulate the expression of osteoblast-specific genes by eliminating reactive oxygen species (ROS) and inhibiting glucocorticoid (GC) treatment–associated side effects (Yang et al., 2016). In addition, TS can increase the expression of osteoprotegerin (OPG) mRNA and type I collagen (CoII-I) mRNA, enhance the activity of alkaline phosphatase (ALP), upregulate the expression level of runt-related transcription factor 2 (Runx2), and promote the formation of calcified nodules (Cui et al., 2009; Han and Wang, 2017). Moreover, TS could suppress oxidative stress–induced osteoporosis and reduce osteoblasts’ apoptosis through the PI3k/Akt pathway (Wang, 2017). In addition, TS can block the NF–κB pathway and inhibit bone cell turnover rates (Han and Wang, 2017). Meanwhile, TS can also regulate osteoclast differentiation and reduce bone resorption through the NF–κB–RANKL signal transduction pathway (Sang, 2019). Additionally, according to evidence from microvascular perfusion imaging of the cancellous bone in GIO rats, as well as the migration capacity of human endothelial cells, TS has a significant protective effect on bone microcirculation. In addition, tanshinol also attenuated GC-elicited the activation of the thioredoxin-interacting protein (TXNIP) signaling pathway and reversed the down-regulation of the Wnt and vascular endothelial growth factor (VEGF) pathways (Lai et al., 2021). Furthermore, another study on glucocorticoid-induced osteoporosis (GIOP) has uncovered that TS could alleviate impaired bone formation. Similarly, co-treatment with TS can effectively offset the impaired bone formation and adipogenesis mechanism caused by oral GC and restore the expression of signal molecules in GIO rats to normal (Yang et al., 2018).

### Limitations

Nonetheless, this study has several limitations. First, although we searched eight databases without any preset restriction on language, it is possible that we may have omitted some relevant studies. Second, given that negative outcomes are not always reported or published, the studies we selected might be biased (supplemental material, Figures 1 and 2). Third, the heterogeneity of tanshinol source and purity cannot be overlooked, as this could jeopardize the validity of our findings. Therefore, additional prospective studies with large sample sizes are warranted to validate our conclusion.

## Conclusion

The study’s main finding indicated that TS treatment could promote BMD, bone microarchitecture, bone biomechanics, and S-OCN expression in rats and that tanshinol, an active component of Salvia miltiorrhiza, has therapeutic potential for treating human OP.

## Data Availability

The original contributions presented in the study are included in the article/Supplementary Material; further inquiries can be directed to the corresponding authors.
